# The healing of bone defects by cell-free and stem cell-seeded 3D-printed PLA tissue-engineered scaffolds

**DOI:** 10.1186/s13018-022-03213-2

**Published:** 2022-06-20

**Authors:** Marjan Bahraminasab, Athar Talebi, Nesa Doostmohammadi, Samaneh Arab, Ali Ghanbari, Sam Zarbakhsh

**Affiliations:** 1 Nervous System Stem Cells Research Center, Semnan University of Medical Sciences, Semnan, Iran; 2 Department of Tissue Engineering and Applied Cell Sciences, School of Medicine, Semnan University of Medical Sciences, Semnan, Iran; 3 Faculty of Metallurgical and Materials Engineering, Semnan University, Semnan, Iran; 4 Faculty of Medicine, Research Center of Physiology, Semnan University of Medical Sciences, Semnan, Iran

**Keywords:** 3D printing, Bone healing, Critical-sized defect, Polylactic acid, Bone marrow-derived mesenchymal stem cells (BMSCs)

## Abstract

In this paper, the in-vivo healing of critical-sized bony defects by cell-free and stem cell-seeded 3D-printed PLA scaffolds was studied in rat calvaria bone. The scaffolds were implanted in the provided defect sites and histological analysis was conducted after 8 and 12 weeks. The results showed that both cell-free and stem cell-seeded scaffolds exhibited superb healing compared with the empty defect controls, and new bone and connective tissues were formed in the healing site after 8 and 12 weeks, postoperatively. The higher filled area, bone formation and bone maturation were observed after 12 weeks, particularly for PLA + Cell scaffolds.

## Introduction

Bony defects in the craniomaxillofacial skeleton due to accidental or surgical trauma are a world wild challenging health concern. Although autologous or allogeneic bone transplantation is a common treatment for bone defects, these have some limitations, such as necrosis, infection, pain, and risk of morbidity; therefore, other alternative methods are needed [1–3]. Tissue engineering and regenerative medicine try to use a combination of bioactive materials, growth factors, and cell therapy to repair bone tissue [4–6]. Due to the osteogenic effect, treatment with cells, especially mesenchymal stem cells (MSCs) may be advantageous in treating critical bone defects caused by severe trauma, osteoporosis, aging, and metabolic diseases like diabetes. The recruitment and migration of MSCs from neighboring tissues to the injured area or defect might not be adequate for differentiation into osteogenic precursor cells in severe bone defects [7]. Therefore, MSCs can be applied more effectively embedded/seeded in/on the scaffold to differentiate into osteo-progenitor cells at the defect site. It has been shown that the seeded MSCs on the scaffold can migrate to the defect site, recruit the source of precursor cells, and finally increase the rate of the scaffold degradation under in vivo conditions. The appropriate biodegradable scaffold assists the migration, adhesion, and proliferation process of stem cells [8–11].

Choosing the suitable material is very important for the manufacture of scaffolds in bone tissue engineering [3, 12]. Three basic characteristics of biomaterials which include bioactivity, biocompatibility, and biodegradability must be considered [13]. Polylactic acid (PLA) is a popular biodegradable polymer with a broad range of applications including medical implant devices, and scaffolds in tissue engineering. The broad use of PLA is due to a combination of favorable properties including its unique biocompatibility, acceptable bioresorbability, generation of nontoxic byproducts during the degradation in the body, and approved clinical trials by the US Food and Drug Administration (FDA) [14]. Furthermore, PLA can be easily printed into scaffolds with different architectures/internal structures and external shapes [15–18]. Therefore, in the current study, PLA was chosen to serve as a template for the delivery of MSCs to the defect site in the rat calvaria. For comparison, the PLA cell-free scaffolds were also implanted and the results were compared. To the authors’ knowledge, there is no study on evaluation of the 3D-printed PLA scaffold along with MSCs in an in-vivo study.

## Materials and methods

### 3D printing of PLA scaffolds

First, a 3D computer-aided design (CAD) model of the PLA scaffold was designed in ABAQUS software. Subsequently, the “stl.” file format of the model was imported to Simplify3D software to provide the g-codes for manufacturing. Poly(lactic) acid filament (diameter of 1.75 mm) was used to build the scaffolds with a conventional fused deposition modeling (FDM) printer. The PLA filament was heated above the PLA melting temperature (nozzle temperature was 210 °C). The melted PLA was extruded through a nozzle made up of stainless-steel on to a printing bed having temperature of 60 °C. The scaffolds were printed in a layer-by-layer manner having a 7.6 mm diameter and a 1.6 mm height. The strut thickness was 0.4 mm and the pore size was 800 μm. The fabricated PLA scaffolds were analyzed using X-ray diffraction (XRD) technique (Bruker, D8-advance) and Attenuated Total Reflection-Furrier Transform Infrared Spectroscopy (ATR-FTIR, Bruker’s Alpha FTIR Spectrometer, Germany). The XRD analysis was conducted at 35 kV and 30 mA using Cu Kα radiation (*λ* = 1.5405980 Å). The scanning angle (2θ) was between 5° and 80° at a step size of 0.06°. ATR-FTIR spectrum was obtained at the resolution of 2 cm^−1^ over the frequency range of 4000–600 cm^−1^. Moreover, the scanning electron microscopy (SEM, Philips XL30, Netherlands) was used to study the morphology (both surface and cross-section) of the 3D-printed scaffolds.

### BMSCs harvesting, culture and immunophenotype

The BMSCs harvesting and culture were done based on the previous study [19]. Briefly, after sacrificing an adult female rat, the femur and tibia were immediately removed. To kill the rat, it was first anesthetized by intraperitoneal (IP) injection of 80 mg/kg ketamine and 10 mg/kg xylazine, and it was followed by cervical dislocation. The bone marrow was flushed by 10 mL of Dulbecco’s Modified Eagle Medium (DMEM, Gibco) with nutrient F12 Ham, supplemented with 10% fetal bovine serum (FBS, Gibco) in two T25 tissue culture flasks, and incubated in the culture medium containing 10% FBS and 1% penicillin/streptomycin at 37 °C, 95% humidity, and 5% CO_2_. After 48 h, the culture medium was replaced. Adhesive cells were sub-cultured four times upon reaching 80–90% confluence.

To analyze the expression of BMSCs surface markers, more than 1 × 10^5^ cells were incubated in fluorescently labeled monoclonal antibodies (BD Pharmingen) against CD29, CD34, CD44, CD45 and CD90 in a dark place. After 30 min of washing with PBS, the labeled cells were analyzed using flow cytometry (BD FACS Calibur). Furthermore, optical microscopy images were taken at different passages for morphology evaluation.

### Sterilizing the PLA scaffolds

The 3D-printed PLA scaffolds were first immersed in distilled water for 1 h. Then, they were washed and ultrasonically cleaned with distilled water for 5 min. Afterward, the cleaned samples were sterilized using ultra-violet (UV) light under a laminar flow bench; 10 min each side of the scaffolds. For the cell-free group, each sterilized PLA scaffold was individually put in a sterile petri dish and transferred for surgery. For the cell-seeded group, the sterilized PLA scaffolds were used to seed MSCs. After seeding (as explained in the following section), each cell-seeded scaffold was individually put in a sterile petri dish with a small amount of complete medium (500µL) and transferred for implantation.

#### BMSCs seeding and culturing on PLA scaffolds

Initially, the bottom of wells of a 24-well plate was evenly coated by 2% agarose (700 μL per well) having no defects including bubbles or scratches in the coatings. After ⁓30 min, the sterilized PLA scaffolds were placed on the agarose. For seeding, 230 µL of culture medium containing 10^6^ BMSCs was added evenly on both sides of the scaffolds; 115 µL on each side. After seeding of the cells on each side of the scaffold, a time interval of about 20 min was considered for initial cell attachment. In the next step, 1 mL of additional culture medium was gently added to each well and the plate was placed in an incubator at 37 °C, with 95% humidity and 5% CO_2_ for 24 h. Finally, the seeded scaffolds were transferred for surgery one-by-one.

Furthermore, the scaffolds 24 h after cell seeding were observed under optical microscope to see the formed cell layer. Images were taken from one cell-seeded scaffold both in live and fixed states. For fixation, paraformaldehyde 4% was used for 15 min at 4 ℃. Further, the fixed sample was studied using SEM (Philips XL30, Netherland).

#### Animals

Wistar female (*n* = 24) adult rats, weighing 250 ± 20 g were used in this study. Animals were kept in a controlled temperature (22 ± 2℃) place and a 12-h regular light/dark cycle (light on from 07:00 to 19:00), housed 2 to 4 per cage with free access to food and water [20]. The experimental protocol was approved by the Ethical Review Board of Semnan University of Medical Sciences (Ethic code: IR.SEMUMS.REC.1400.048). All experiments were conducted in agreement with the National Institutes of Health Guide for the Care and Use of Laboratory Animals.

#### Implantation

**Fig. 1:**
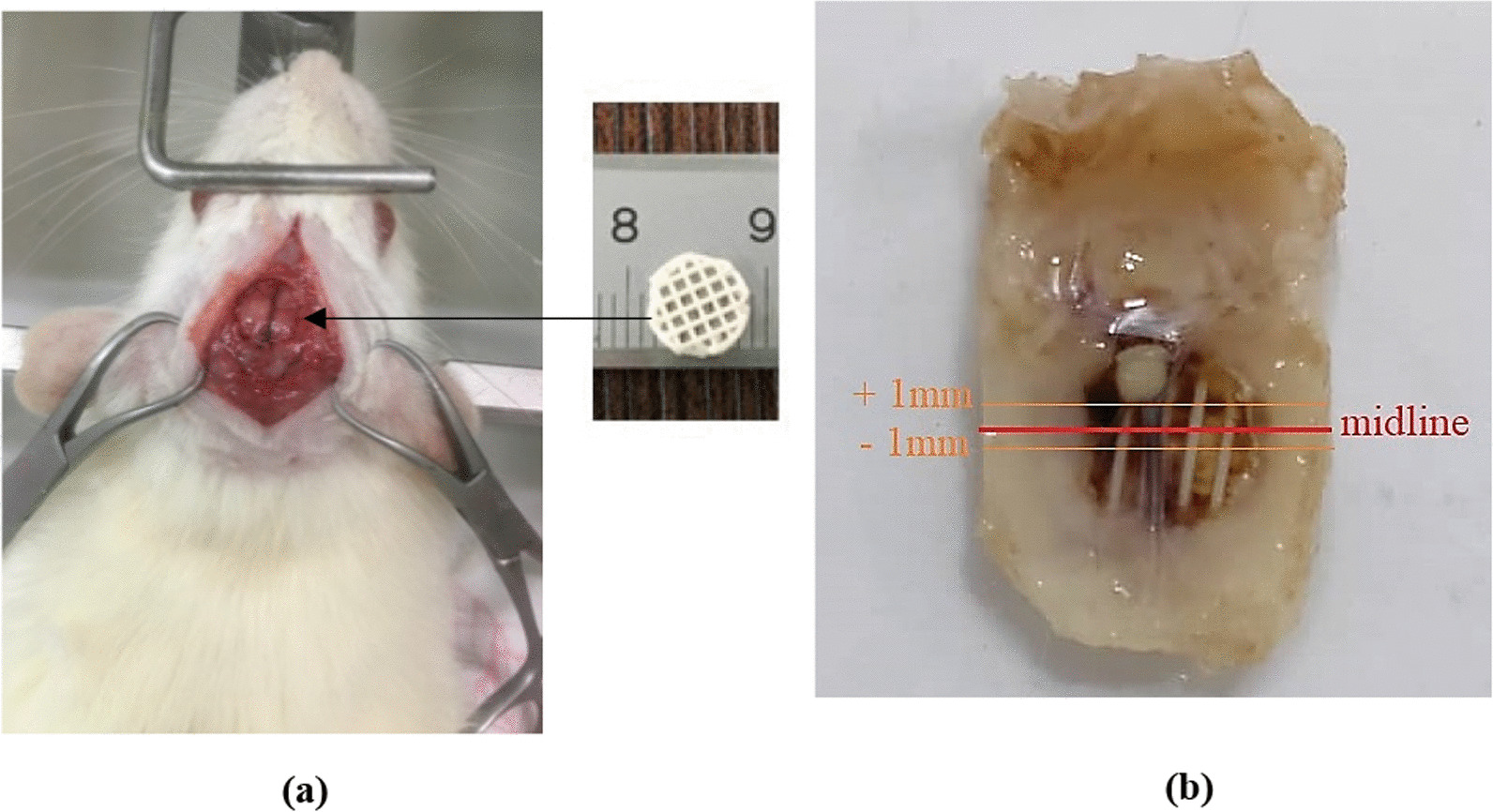
**a** Drilled hole and 3D-printed scaffold, and **b** analyzed area in histology

The scaffold implantation was performed through a method which was described by Sadeghi et al. [21]. After anesthetizing of the rats by IP injection of a mixture containing ketamine hydrochloride and xylazine hydrochloride with the volume ratio of 8:2 at 1 mL/Kg [22], the head of the rat was completely fixed in a stereotaxic apparatus, and the hair was shaved and the skull was disinfected using povidone iodine solution. To expose the full extent of the calvaria, subperiosteal dissection was done bilaterally in a non-infectious manner and the subcutaneous muscles were completely pushed away. The skull was drilled to the size of the scaffold using a surgical trephine bur. One calvaria through-and-through osteotomy was made in the dorsal portion of the parietal bone midsagittal suture (Fig. 1a) under irrigation with sterile normal saline. After preparing the transplant conditions, the scaffold was placed into the hole and finally, the scalp was sutured. The bone repair was analyzed after 8 and 12 weeks, postoperatively. In this study, 3 groups were investigated; (1) control group which were the rats having defects without treatment (no scaffolds), (2) PLA group in which the defects were implanted by PLA scaffolds (without stem cells considered as cell-free), and (3) PLA + Cell group in which the defects were implanted by stem cell-seeded PLA scaffolds. The number of animals in the PLA (without cells) and PLA + Cell, groups were 5 in each time interval (totally 20 rats) and for control group (defect without treatment) the number of animals were 2 in each time point (totally 4 rats). The details of animal groups are given in Table 1.

**Table 1:** Details of studied animal groups

Group number	Group name	Time (week)	Number of implanted rats for each implantation time
1	Defect	8	2
		12	2
2	PLA	8	5
		12	5
3	PLA + Cell	8	5
		12	5

### Histological analysis

**Fig. 2:**
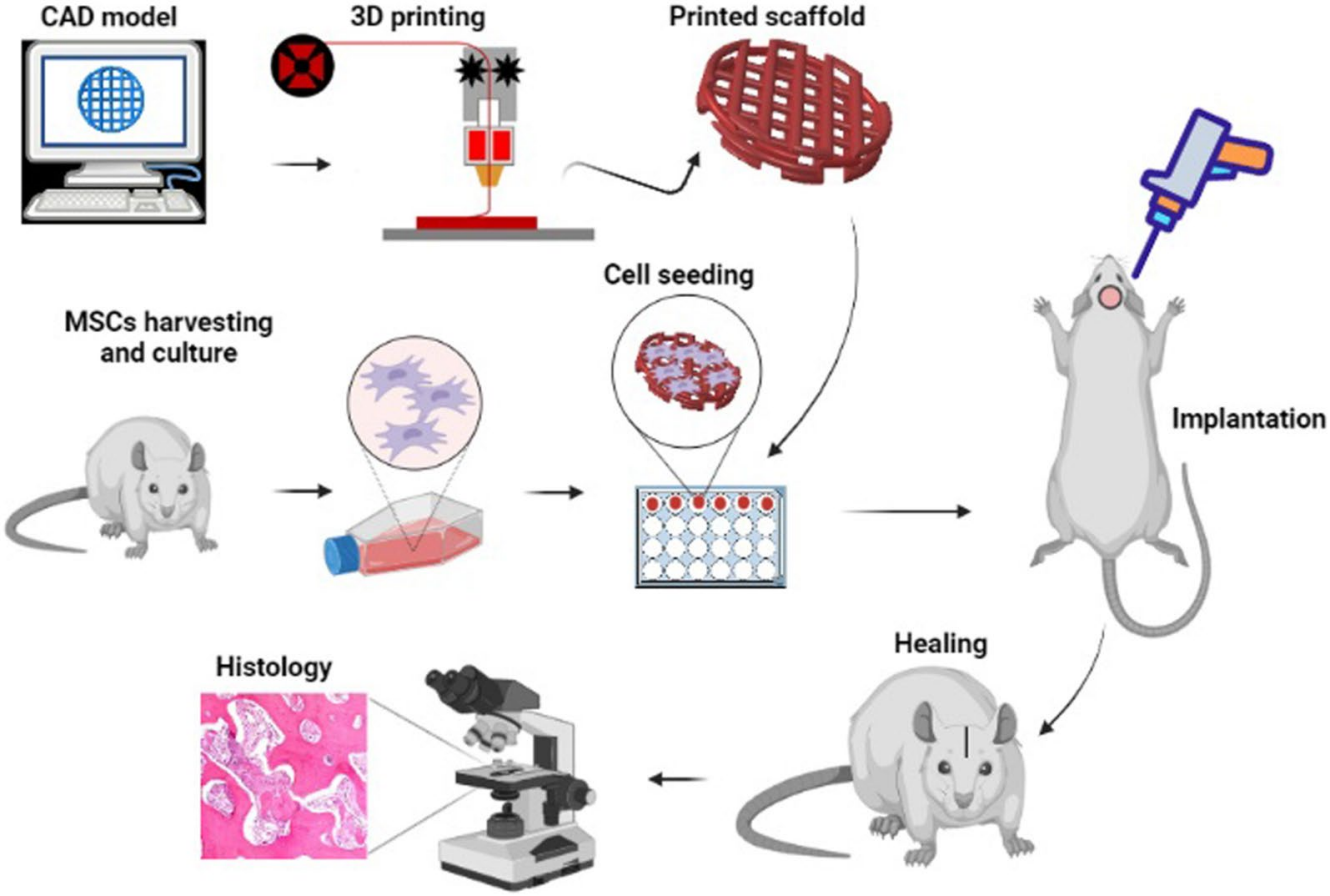
The general steps used in this study

The histological analysis was conducted after 8 and 12 weeks. First, the rats were sacrificed and then the defect sites were judiciously dissected. These samples were fixed in neutral-buffered formalin (10%), and decalcified in formic acid (10%), sequentially. The standard dehydration was then conducted on the decalcified samples in serially increasing alcohol (ethanol) solutions. The dehydrated samples were embedded in paraffin, and subsequently 5 µm sections were provided. The prepared sections were stained with hematoxylin and eosin (H&E). The analyzed area in histology is shown in Fig. 1b. Some sections were also stained by toluidine blue. A light microscope was employed to analyze the histology slides. The percentages of the new bone area and the number of blood vessels were measured from H&E images using ImageJ software. To calculate the new bone percentage, the area of newly formed bone was divided to the whole area of the new tissue in each image and expressed in percent. Furthermore, immediately after harvesting the defect sites, photos were taken and the macroscopically filled area by new tissue was calculated in percent using ImageJ software. Figure 2, shows the whole procedure used in this study.

### Serum biochemistry and osteocalcin detection

To assess the systemic toxicity of the scaffolds, the level of liver and muscle enzymes was measured by serum biochemistry. At the time of sacrifice (8 or 12 weeks postoperatively), about 5 mL of blood was collected from the heart of each rat and centrifuged at 3000 rpm for 10 min to obtain blood serum. Alanine aminotransferase (ALT) and aspartate aminotransferase (AST) were analyzed using the commercial kits (Paadco, Golestan Technology Park, Iran). Furthermore, the osteocalcin level (a bone formation marker) was also measured in the serum, using a sandwich ELISA method (Rat Osteocalcin/Bone Gamma-Carboxyglutamic Acid Containing Protein (OT/BGLAP) EISA Kit; ZellBio, Germany) according to the manufacturer instruction.

### Statistical analysis

Analysis of Variance (ANOVA) was done for statistical analyses using Minitab V17 software. The confidence level was set to be 95% (*α* = 0.05) in all analyses. Moreover, the post-hoc pairwise comparisons were conducted using Tukey test.

## Results

### Phase analysis and chemical bonds

**Fig. 3:**
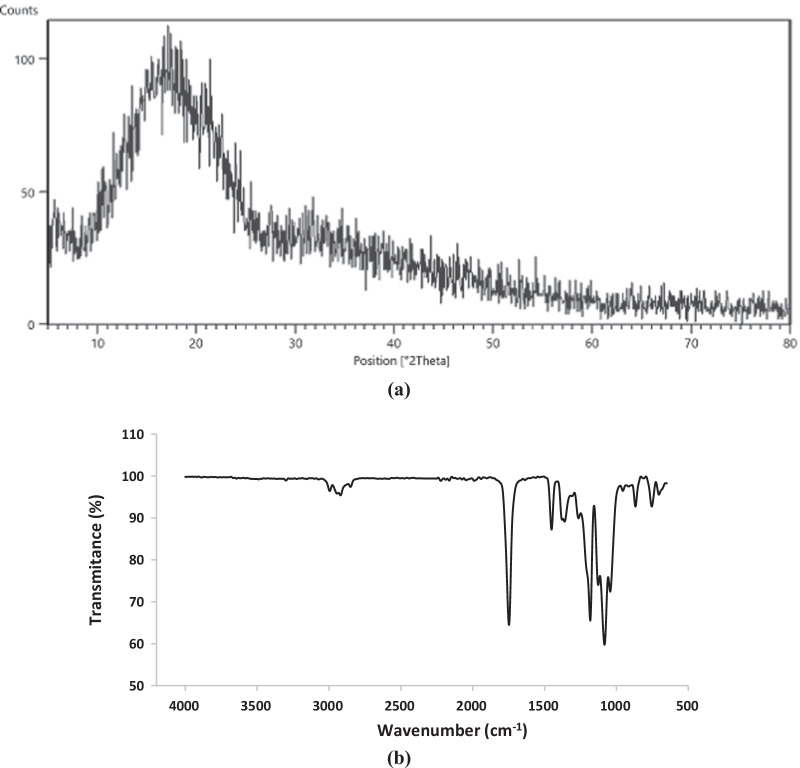
**a** XRD, and **b** ATR-FTIR spectra of 3D-printed PLA scaffold

Figure 3a shows a noisy background with no sharp and narrow diffraction peaks in PLA indicating its amorphous nature. There is a large broad peak extending from 2*θ* of around 10° to < 30°. Two small peaks also can be seen at 5° < 2*θ* < 10° and 30° < 2*θ* < 35°. The obtained pattern corresponds to the characteristic peaks of PLA [23–26]. The ATR-FTIR spectra of PLA scaffold is shown in Fig. 3b. The characteristic bands at about 2994 and 2944 cm^−1^ are related to –CH stretching in –CH_3_ group. The peak appeared at 2922 cm^−1^ is attributed to –CH bending vibration. Furthermore, some peaks were also observed at 756 and 867 cm^−1^ (–CH bending vibration); 1043 cm^−1^ and 1182 cm^−1^ (C–O stretching vibration); 1082 cm^−1^ (stretching peaks of C–O–C bonds); 1451 cm^−1^ and 1361 cm^−1^ (–CH bending in –CH_3_ group); and 1748 cm^−1^ (C=O stretching vibration on ester group)**.** The observed peaks correspond to PLA and agree well with the data in the previous studies [27–29]**.**

### BMSCs morphology and immunophenotype

**Fig. 4:**
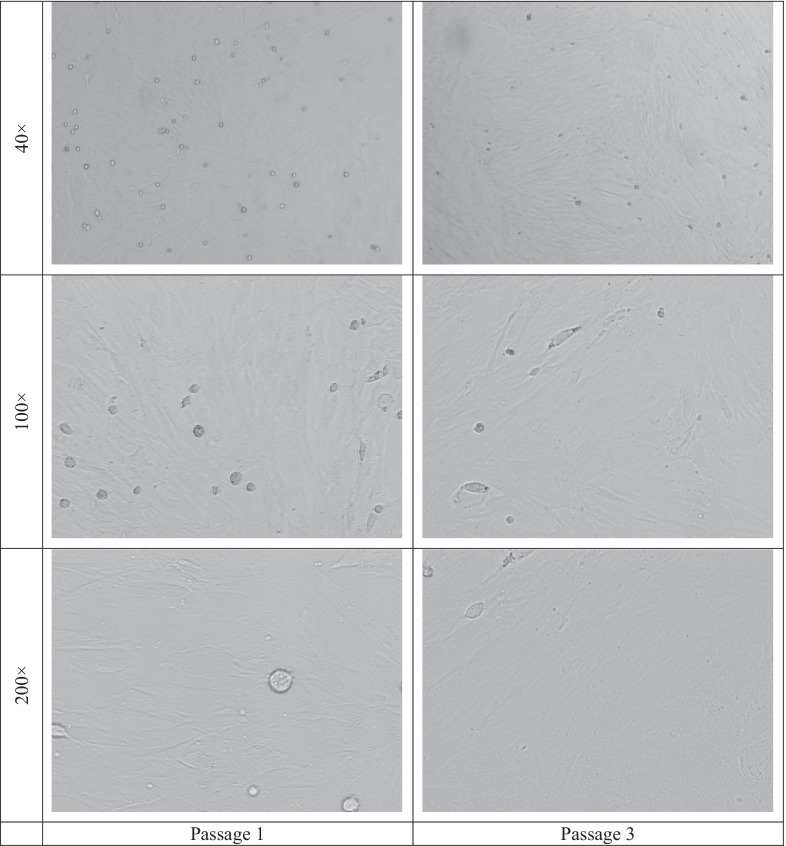
The morphology of BMSCs during culture

**Fig. 5:**
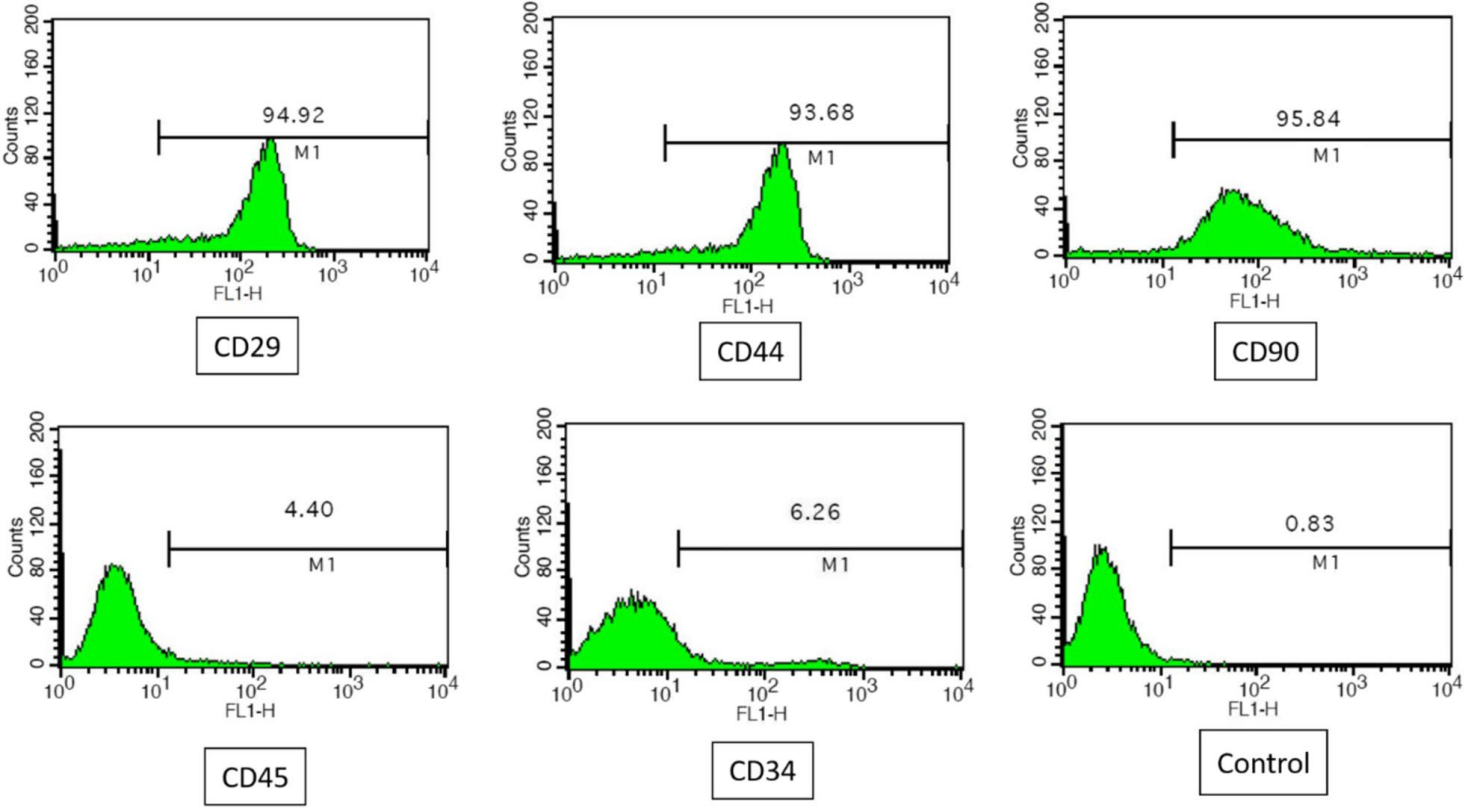
Flow cytometry analysis of BMSCs immunophenotype

The morphology of the BMSCs was observed during culturing which was appeared to be normal; adherent in spindle shape with fibroblastic morphology [30, 31]. Figure 4 shows the morphology of BMSCs during culture (passages 1 and 3). The flow cytometry analysis illustrated that BMSCs isolated from rat bone marrow were positive for the cell surface markers CD29 (94.92%), CD44 (93.68%), and CD90 (95.84%), while were negative for CD34 (6.26%) and CD45 (4.40%) (Fig. 5). These results suggested that the cultured cells had similar morphological and immunophenotypical characteristics to BMSCs.

### Seeded BMSCs on scaffolds

**Fig. 6:**
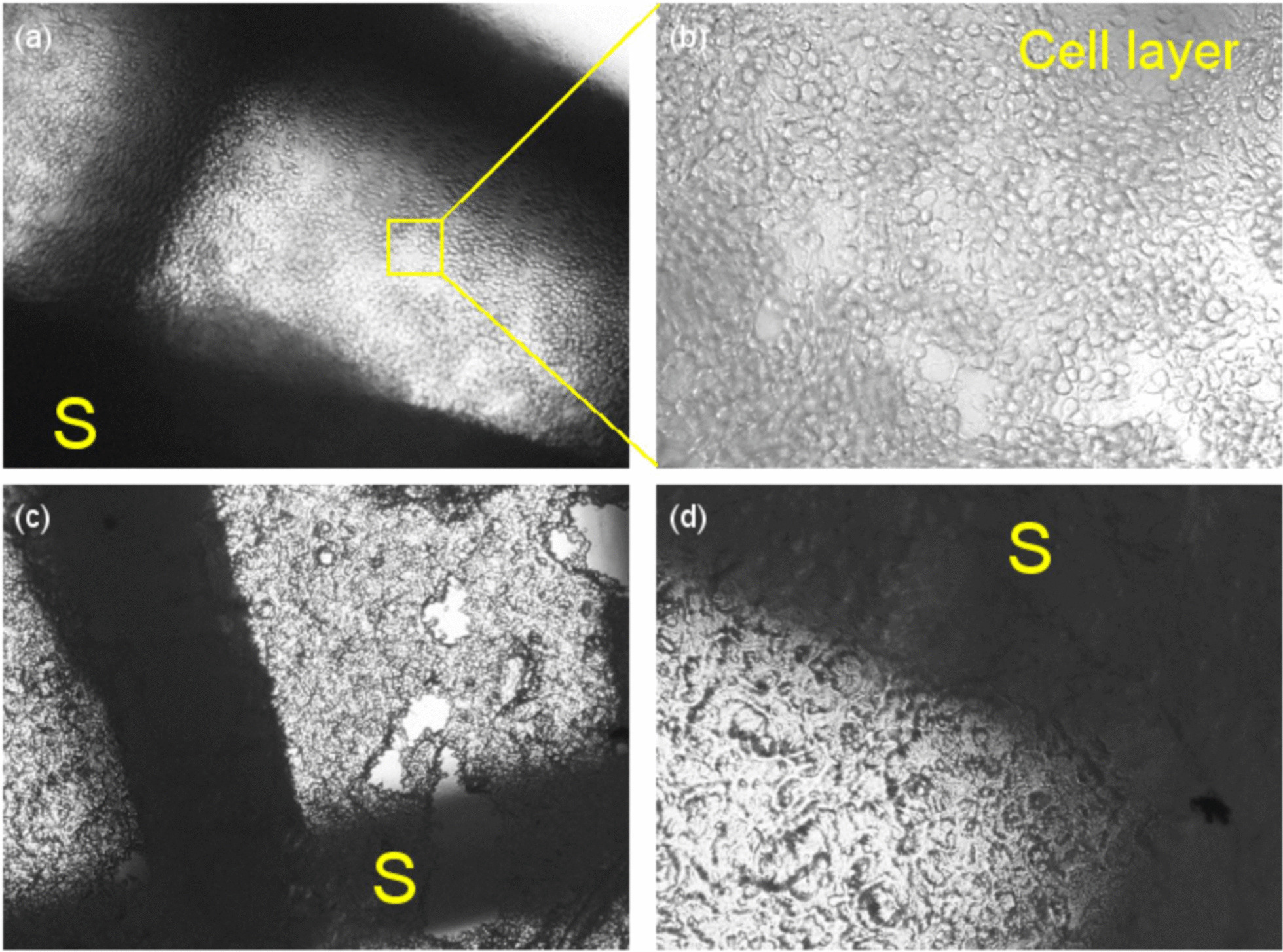
Cell layer on the scaffold; **a** and **b** live cells, and **c** and **d** fixed cells. The magnifications were 40 × and 100 × for **a** and **c** and **b** and **d**, respectively. The scaffold is shown by “S”

**Fig. 7:**
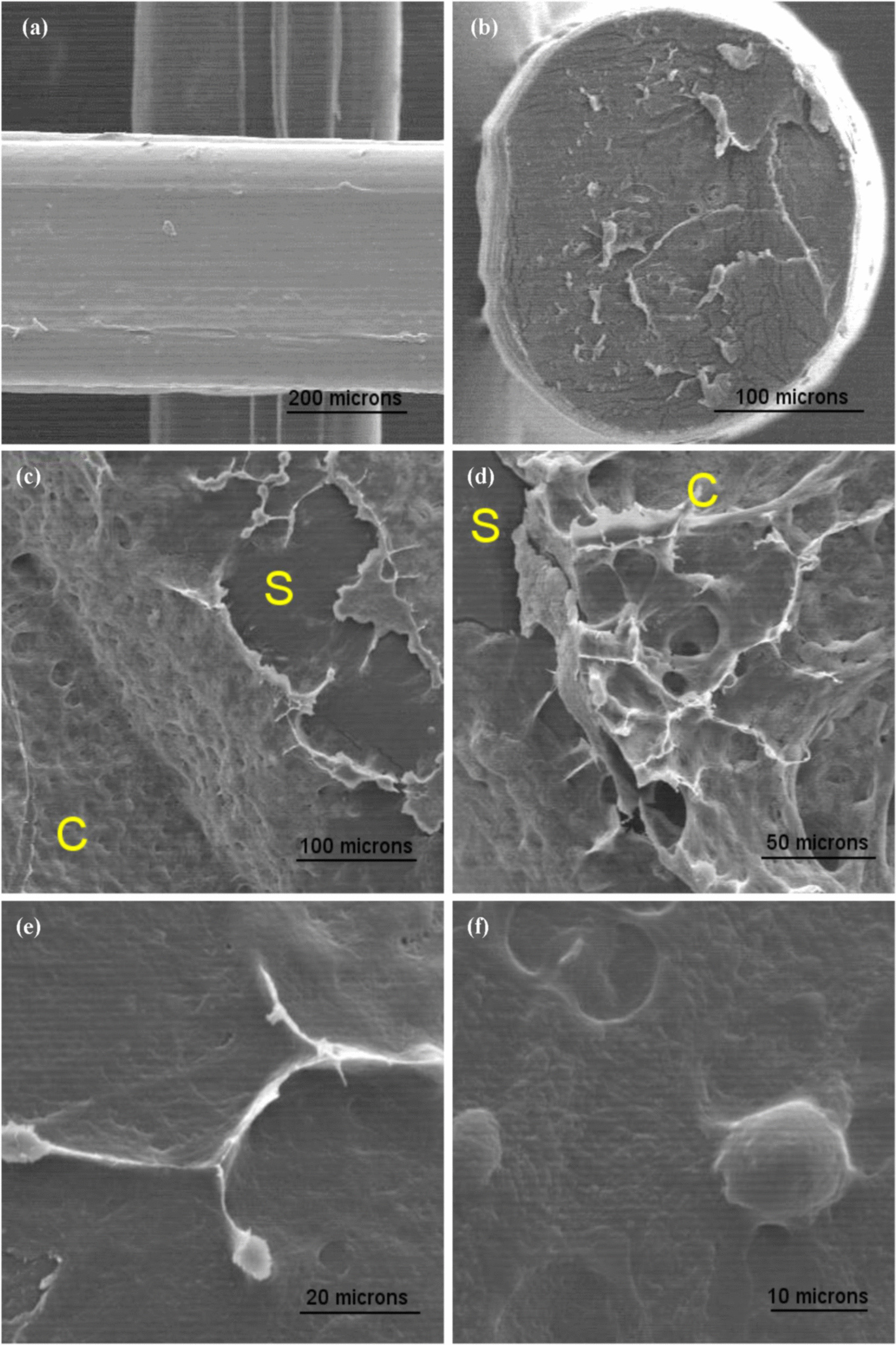
SEM images of **a** PLA scaffold, **b** scaffold cross-section, and **c–f** fixed cell layer on the scaffold. The scaffold and cell layer are shown by “S” and “C,” respectively

Immediately, after cell seeding (live cells) as well as after fixation, the PLA scaffolds were observed under an optical microscope. As it can be seen in Fig. 6, a dense cell layer was seeded on the scaffolds. Furthermore, SEM images were taken from the surface and cross- section of a PLA scaffold (Fig. 7a, b), and from the surface of the scaffold with fixed cells (Fig. 7c–f). In these figures, the scaffold is shown by “S” and the cell layer is indicated by “C.”

### Histology and histomorphometry analysis

**Fig. 8:**
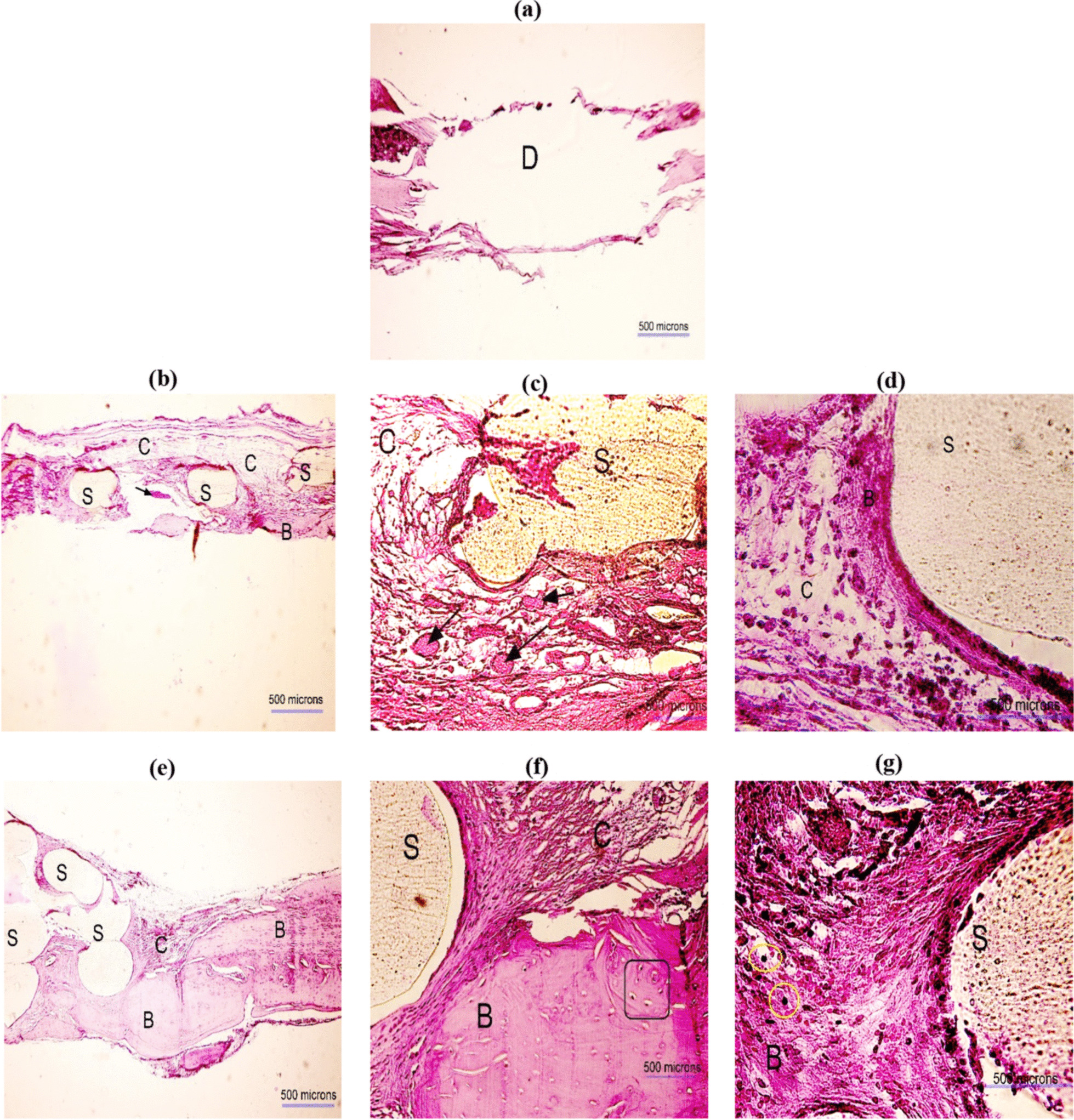
H&E images obtained 8 weeks postoperatively in different groups; **a** defect without scaffold, **b–d** PLA scaffold, and **e–g** PLA + Cell scaffold. The scaffold, bone, connective tissues, and defect site are shown by “S,” “B,” “C,” and “D,” respectively. The new bone islands are indicated by black arrows and osteocytes are identified in rectangles. The magnifications are ×40, ×200, and ×400 in **a**, **b** and **e**, **c** and **f**, and **d** and **g**, respectively

**Fig. 9:**
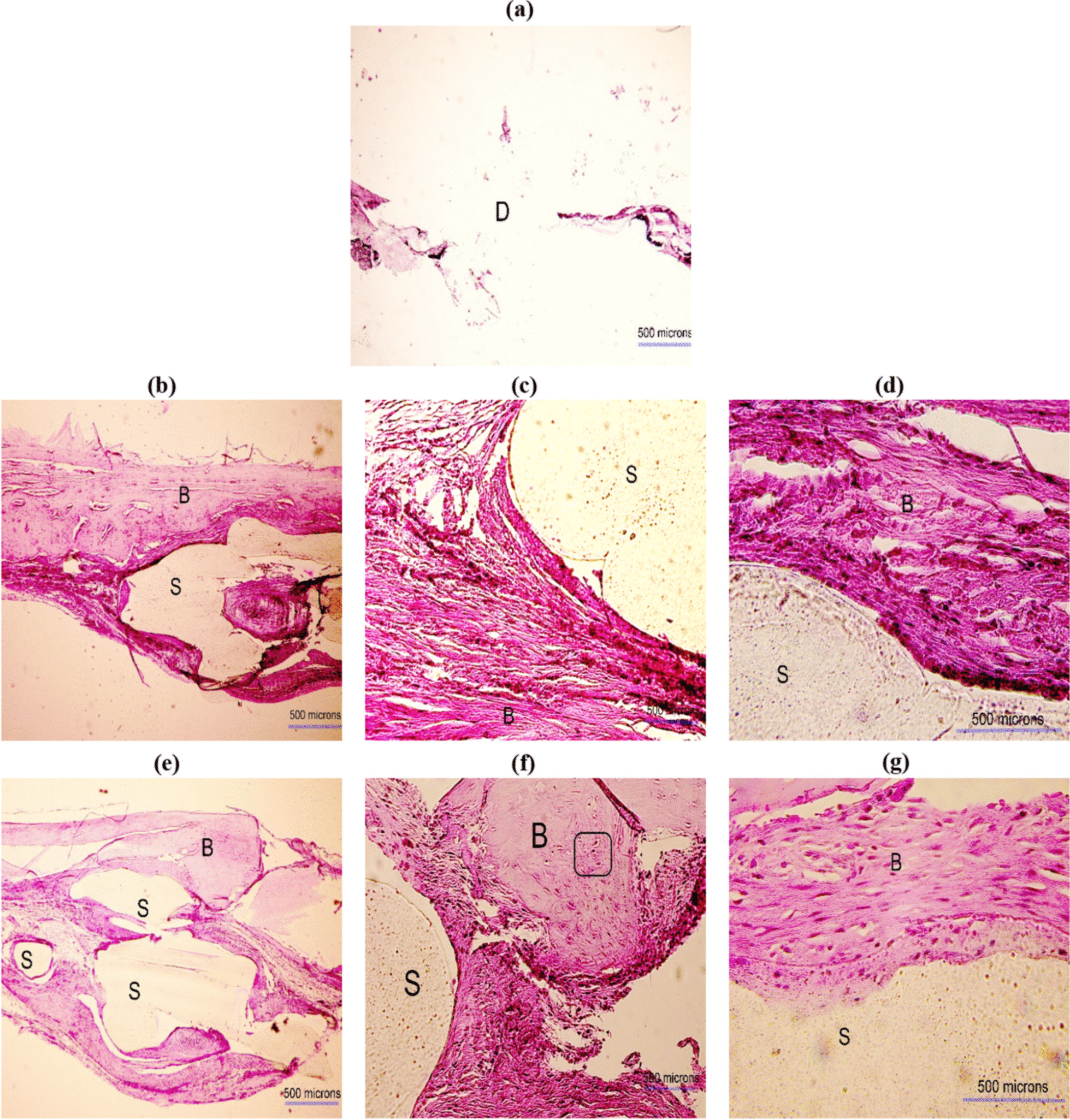
H&E images obtained 12 weeks postoperatively in different groups; **a** defect without scaffold, **b–d** PLA scaffold, and **e–g** PLA + Cell scaffold. The scaffold, bone, connective tissues, and defect site are shown by “S,” “B,” “C,” and “D,” respectively. The magnifications are ×40, ×200, and ×400 in **a**, **b** and **e**, **c** and **f**, and **d** and **g**, correspondingly

Figures 8 and 9 show the histological H&E images taken 8 and 12 weeks after implantation, respectively. In these figures, the scaffold is shown by the letter “S” (light beige color), the defect is identified by the letter “D,” and the bone is marked by the letter “B” (pink color). The osteocytes are indicated in rectangles and the new bone islands are pointed at by arrows. The scaffolds still remained at the defect site meaning that the biodegradability of the PLA scaffold was slow. However, the new tissues including connective, and neo-bone were formed around the scaffold struts in the pores. In the PLA groups (cell-free) more connective tissues and collagen fibers, and smaller bone, particularly in the lamellar form were observed. However, these were more obvious at week 8 rather than week 12. In the PLA + Cell groups, more blood vessel formation and a small number of lymphocytes (yellow circle in Fig. 8) were observed. A higher degree of new bone formation, more lamellar bone and cartilaginous tissue were observed in the PLA + Cell groups compared with cell-free PLA scaffolds. As it can be seen in Figs. 8a and 9a, no tissue formation was seen in the untreated defect groups.

**Fig. 10:**
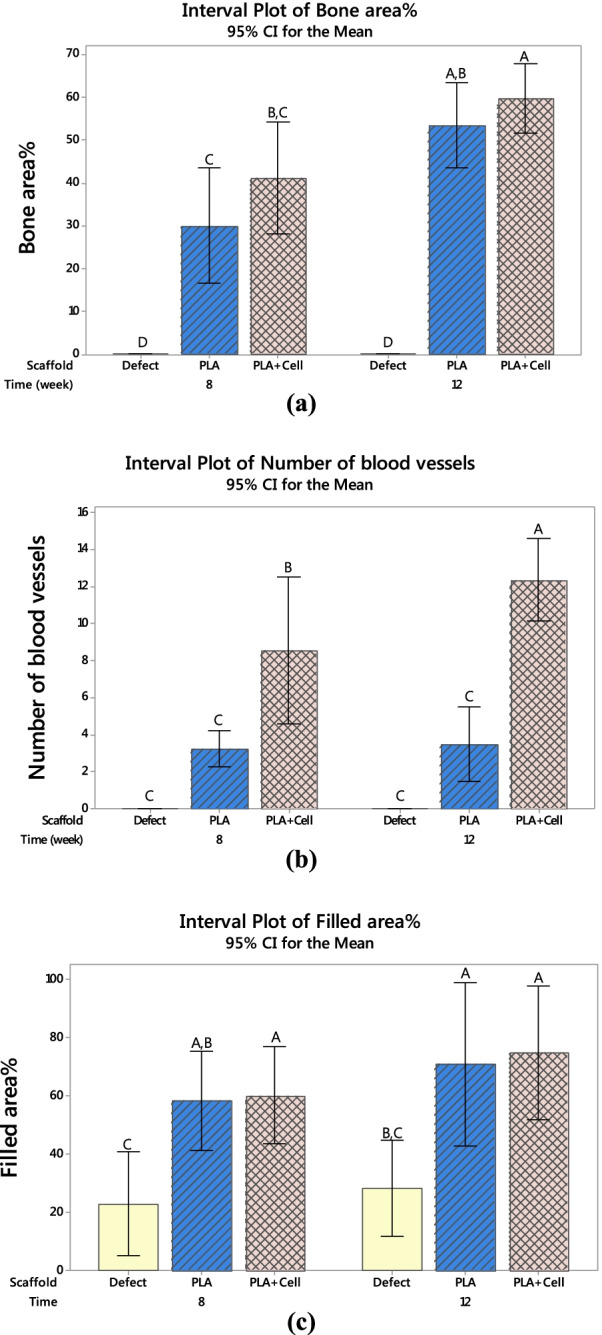
Comparison of **a** bone area%, **b** number of blood vessels, and **c** filled area% in different groups (the means that do not share a letter are significantly different)

Figure 10a shows the percentages of the bone area formed in the defect site around the scaffolds. The mean values of bone area% for PLA (cell-free) and PLA + Cell were 30.0, 41.2, 53.5, and 59.8% after 8 and 12 weeks, respectively. In addition, a higher new bone area was observed in the defect sites after 12 weeks in both groups. The ANOVA results revealed that the implantation time, the scaffold type, and their interaction were significant factors on bone area formed (*P*-value = 0.000, *P*-value = 0.000, and *P*-value = 0.032) at the confidence level of 95%. In Fig. 10, the means that do not share a letter are significantly different (results of Tukey test). Therefore, the new bone area formed around PLA scaffolds after 8 weeks was significantly lower than PLA and PLA + Cell scaffolds after 12 weeks. Meanwhile, the bone area% in the defect site around PLA + Cell scaffolds after 8 weeks was significantly different from that after 12 weeks. However, there were no statistically significant differences between PLA and PLA + Cell scaffolds after 8 weeks as well as after 12 weeks. Figure 10b shows the number of blood vessels formed around the PLA (cell-free) and PLA + Cell scaffolds. As it can be obviously seen, the number of vessels was significantly higher in PLA + Cell groups than the PLA groups, particularly after 12 weeks. The statistical analysis also indicated that the only significant factor in blood vessel formation was the scaffold type (*P*-value = 0.000). Figure 10c represents the filled area of the defect by new tissue in the studied groups. The values in this figure were normalized to the defect size at the operation day. As it can be seen the filled area increased in all groups at week 12 compared to week 8. Furthermore, the filled area was highest for the PLA + Cell group, 12 weeks postoperatively. The results of ANOVA also indicated that the scaffold is a significant factor in filled area% (*P* value = 0.000). The Tukey pairwise comparisons indicated that the areas of the defect filled by new tissue (both soft and hard tissues) were significantly higher in PLA (cell-free) and PLA + Cell group compared with the untreated control group (defect without scaffold). It should be pointed out that in the untreated group the filled area of the defects was mostly non-functional soft tissue, while in the scaffold groups, as indicated by H&E staining, the filled area mainly had cartilaginous and bone tissues.

**Fig. 11:**
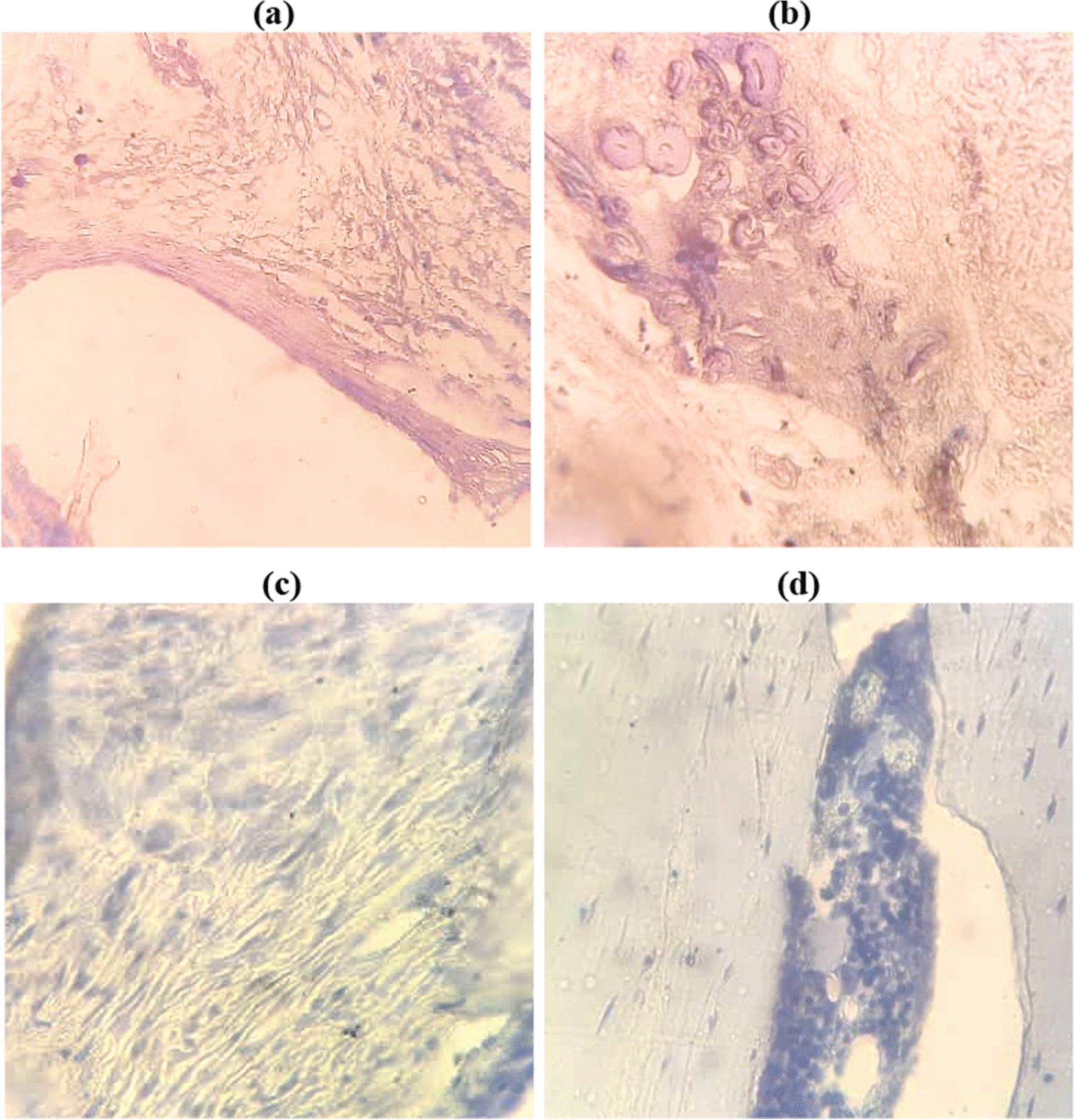
Toluidine blue staining obtained 8 weeks postoperatively in (a and b) PLA group, and (c and d) PLA + Cell group (× 400)

**Fig. 12:**
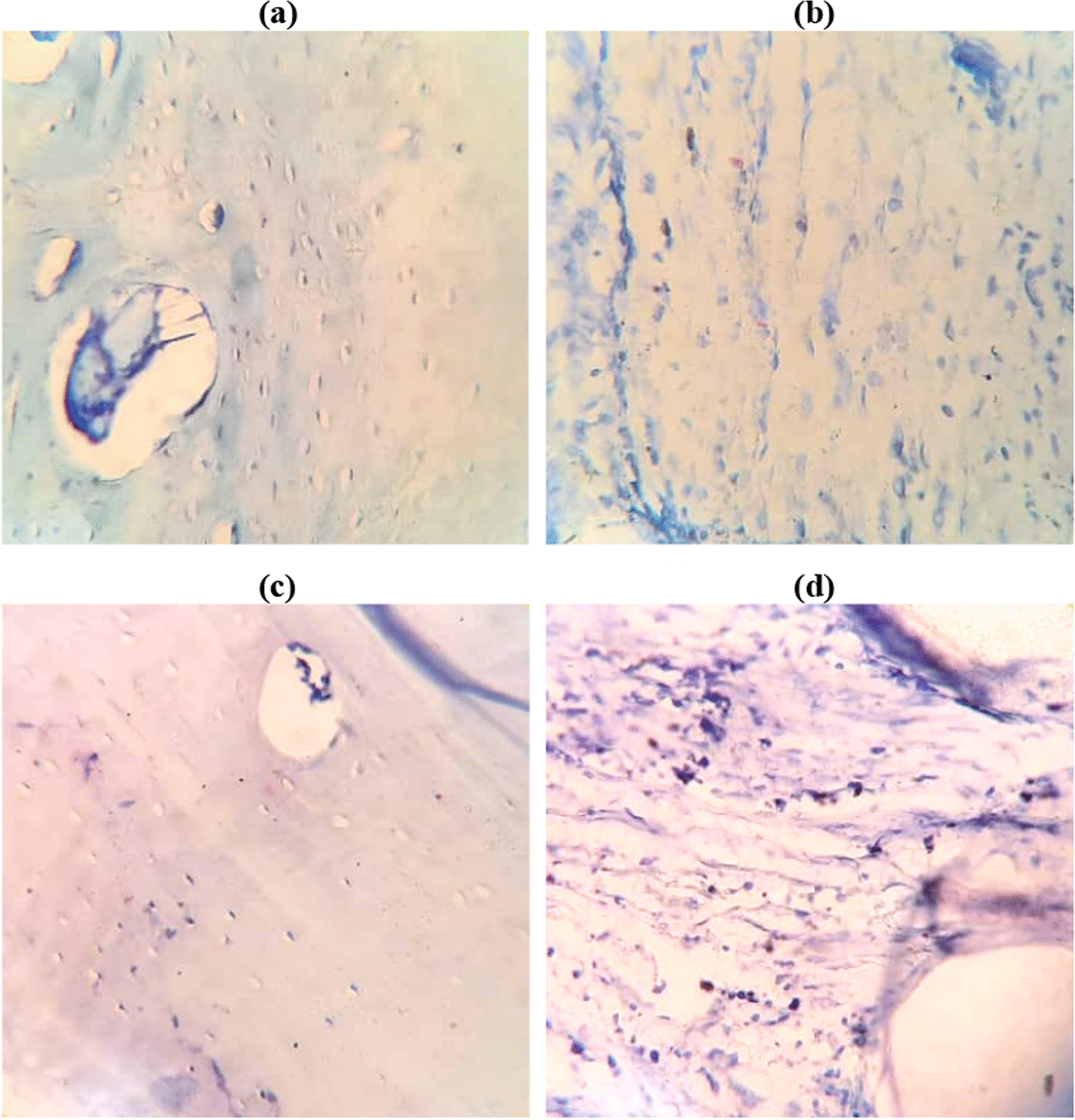
Toluidine blue staining obtained 12 weeks postoperatively in **a** and **b** PLA group, and **c** and **d** PLA + Cell group (× 400)

Toluidine blue, a cationic dye, stains the proteoglycans as well as glycosaminoglycans in the tissue [32]. Proteoglycans are one of the most abundant constituents of the non-collagenous proteins in the bone matrix. These are characterized by the covalent bond of long-chain polysaccharides (glycosaminoglycans) to core protein molecules [33]. Therefore, toluidine blue stains the tissue where bone matrix and connective tissue are formed. Figures 11 and 12 show the toluidine blue staining of the tissues 8 and 12 weeks after implantation. As it can be seen in these figures, in the PLA + Cell groups both new bone and connective tissue were clearly observed. These features were also seen in the PLA group at week 12. However, at week 8, the area of the colored tissue was very small as shown in Fig. 11a and b. This is in agreement with the results obtained in H&E staining.

### ALT, AST, and osteocalcin level

**Fig. 13:**
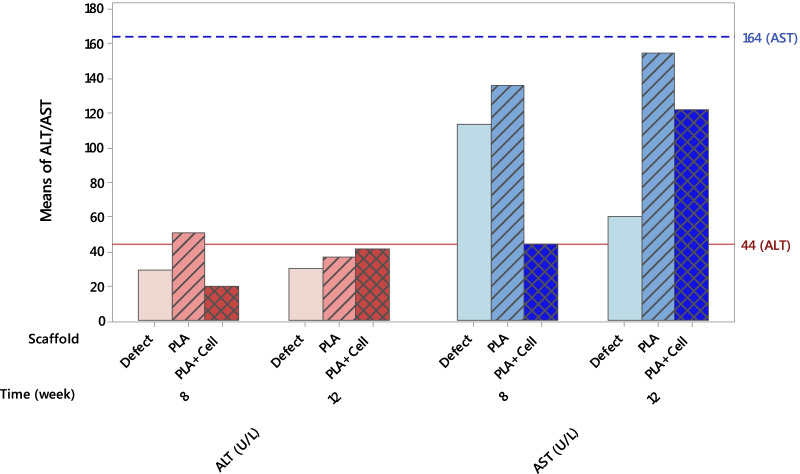
ALT and AST level in rat serum 8 and 12 weeks postoperatively in different groups

The biochemical analyses (ALT and AST level) were performed to monitor the systemic influence and any abnormal response possibly induced by the scaffolds. AST and ALT are sensibly sensitive indicators of liver injury or damage from various types of diseases or conditions. The ALT and AST levels in rat serum of different groups are shown in Fig. 13. The continuous red line and dashed blue line in Fig. 13 indicate these enzyme levels for normal female rats with neither skull defects nor scaffold implantation. The AST levels in all groups of scaffolds did not exceed the dashed blue line meaning that the scaffolds did not cause systemic toxic effects. Similarly, the ALT levels did not surpass the normal level except for the rats with PLA scaffold implantation after 8 weeks. However, after 12 weeks the ALT value decreased below the reference line. Furthermore, there were no significant differences between groups in ALT and AST levels.

The serum osteocalcin levels (Table 2) were higher at week 8 than week 12 in all groups. The highest osteocalcin level was associated with PLA + Cell at week 8. The level of this osteoblastic marker was lower at week 12 possibly due to the fact that after this time the defects were mostly filled by new bone. The level of osteocalcin in the PLA + Cell group at week 12 was significantly different from PLA + Cell and PLA at week 8, and PLA at week 12. Nevertheless, the level of osteocalcin in the defect group was not changed at weeks 8 and 12. This is due to the fact that no bone matrix was formed at the defect site in this group.

**Table 2:** Osteocalcin levels in rat serum of studied groups

Group	Time (weeks)	Osteocalcin (ng/mL)
Defect	8	2.196 ± 0.147
	12	2.136 ± 0.059
PLA	8	2.092 ± 0.056
	12	2.031 ± 0.022
PLA + Cell	8	2.417 ± 0.084
	12	1.792 ± 0.050

## Discussion

In the present paper, the efficacy of 3D-printed PLA scaffolds both as cell-free and cell-seeded construct was assessed in the repair of a critical-sized defect in rat calvaria. Our findings suggest that both scaffolds provide efficient templates for new bone growth and repair without producing any toxic effects. The ALT and AST levels in blood serum are indicative of the systemic influence of the implanted scaffolds on liver function [34–36]. In the results obtained here, the levels of these two enzymes laid below the normal levels. PLA is a synthetic polymer that is widely used in tissue engineering applications and its high biocompatibility has been reported frequently [37–40]. Our findings also rejected any toxicity or abnormality caused by neither scaffold material nor the manufacturing approach.

The osteocalcin concentration was measured in blood serum. Osteocalcin is produced only by mature osteoblasts and plays a role in bone mineralization [41]. It is mainly deposited into the bone extra cellular matrix (ECM) and only a small quantity of its newly formed reaches the circulation [42, 43]. The highest osteocalcin level in PLA + Cell at week 8 might indicate the higher mineralization in this group (as confirmed by the result shown in Fig. 10a). The level of this osteoblastic marker at week 12 was lower in all groups and it may be due to the fact that after this time the defects were mostly filled by new bone. In one study conducted by Zhang and Zhang [43], the osteocalcin expression by MG63 exposed to microporous chitosan scaffolds reinforced by calcium phosphate was assessed. The authors obtained lower osteocalcin concentration at day 11 compared with that of day 7. Their result on reduction of osteocalcin level by time is in agreement with our findings. Another point is that the level of this osteoblastic marker in the defect group was not changed at different time points. This is due to the fact that no bone matrix was formed at the defect site in this group.

Both PLA and PLA + Cell scaffolds showed to induce tissue regeneration at the defect site. The connective and bone tissues along with collagen fibers and blood vessels were formed around the scaffold struts. However, the histological analysis revealed that the PLA + Cell scaffolds caused better bone formation and repair. The presence of BMSCs on the scaffold appeared to help in the bone regeneration process. MSCs are known as self-renewing, multipotent cells, which exist in different body tissues and are considered as reparative cell reservoirs. These cells differentiate in response to signaling at the site of injury [44, 45]. Furthermore, MSCs can contribute to the maintenance of stem cell niche and tissue homeostasis [46]. Moreover, they have low immunogenicity and show effective immune-suppressive qualities. Nevertheless, the MSCs recruitment and migration from adjacent tissues to the defect site is not probably adequate for differentiation into osteogenic precursor cells in severe bone defects [7], such as the critical-sized defect (7.6 mm) in the present study. Therefore, scaffolds can be employed to have a more effective migration of the MSCs differentiating into osteo-progenitor cells at the defect area. The promising results of our histological analysis suggest that the PLA scaffold provided an appropriate environment for the viability of the BMSCs. This can be attributed to the scaffold structural characteristics including proper biomaterial composition, porosity percentage, and pore sizes [8, 47–49]. These properties along with mechanical stability, stiffness, biodegradation, and non-toxicity are required for the successful performance of an implanted scaffold [50, 51]. PLA has this combination of properties which results in an acceptable function in-vivo. The only drawback of PLA is its hydrophobicity and reduced cell adhesion [52]. Therefore, some studies focused on PLA modification using bioceramics or surface treatments [53, 54]. It would be interesting to study the in-vivo performance of the treated PLA and its composites along with BMSCs to find out about the synergistic and antagonistic effects. Another issue that can be considered in future studies is the use of growth factors that can provide the proper signaling and help in stem cell differentiation [55].

## Conclusions

The results from this study showed the two scaffolds can encourage effective healing of a critical-sized defect in rat calvaria compared to the untreated controls (empty defects without scaffolds). The 3D-printed porous PLA scaffold was a suitable framework for BMSCs seeding as they could differentiate to bone cells and contribute to the healing. According to the results obtained here, the osteogenesis of the 3D-printed PLA scaffold were enhanced after loading it with the BMSCs. Therefore, the scaffold has the potential for future bone tissue engineering applications.

## Data Availability

The data will be available upon request.

## Abbreviations

3D: Three dimensional
ALT: Alanine aminotransferase
ANOVA: Analysis of variance
AST: Aspartate aminotransferase
ATR-FTIR: Attenuated total reflection-furrier transform infrared spectroscopy
BMSCs: Bone marrow-derived mesenchymal stem cells
CAD: Computer-aided design
DMEM: Dulbecco’s modified eagle medium
ECM: Extra cellular matrix
FDA: Food and drug administration
FDM: Fused deposition modeling
H&E: Hematoxylin and eosin
IP: Intraperitoneal
MSCs: Mesenchymal stem cells
PLA: Polylactic acid
SEM: Scanning electron microscopy
UV: Ultra violet
XRD: X-ray diffraction
